# A Practical Approach to Evaluate Lattice Thermal Conductivity in Two-Phase Thermoelectric Alloys for Energy Applications

**DOI:** 10.3390/ma10040386

**Published:** 2017-04-05

**Authors:** Yaron Amouyal

**Affiliations:** Department of Materials Science and Engineering, Technion–Israel Institute of Technology, 32000 Haifa, Israel; amouyal@technion.ac.il

**Keywords:** thermal conductivity, thermoelectric materials, first principles calculations, vibrational properties, lead−telluride-based compounds

## Abstract

Modelling of the effects of materials’ microstructure on thermal transport is an essential tool for materials design, and is particularly relevant for thermoelectric (TE) materials converting heat into electrical energy. Precipitates dispersed in a TE matrix act as phonon-scattering centers, thereby reducing thermal conductivity. We introduce a practical approach to tailor a definite precipitate size distribution for a given TE matrix, and implement it for PbTe. We evaluate vibrational properties from first principles, and develop an expression for phonon relaxation time that considers both matrix vibrational properties and precipitate size distribution. This provides us with guidelines for optimizing thermal conductivity.

## 1. Introduction

Heat transport phenomena play significant roles in many technological applications [1]. Modelling of the effects of microstructure on thermal transport in multiphase materials is of utmost importance, since it provides us with practical knowledge concerning materials selection and materials design issues, which are involved in specific engineering demands. Two extreme examples for this are materials design for heat removal, such as for heat sinks and radiators, in which high thermal conductivity is demanded; alternatively, design of materials for thermal insulation requires materials with low thermal conductivity [1]. Either way, heat transport phenomena play critical roles, and are sensitive to the finest features in the materials’ microstructure, such as the presence of particles/second-phase precipitates, internal interfaces, dislocations, alloying elements, or any other point defects [2,3,4,5,6].

Development of thermoelectric (TE) materials is one of the most prominent examples of the correlation between microstructure and thermal properties. TE materials are able to convert heat flux into electrical current via the *Seebeck* effect, or vice versa, via the *Peltier* effect [7,8,9,10]. Such materials are, therefore, essential for electrical power generation from waste heat or for refrigeration by heat pumping [11]. Besides having an intrinsically large Seebeck coefficient (*S* is defined as the open-circuit voltage, ΔV, produced as a result of a temperature difference, ΔT, between the two poles: $S=\frac{\Delta V}{\Delta T}$), a good TE material should possess high electrical conductivity, σ, and low thermal conductivity, κ; all are embodied in the dimensionless TE *figure of merit*:

(1) $$ZT=S^{2}T\frac{\sigma }{\kappa }$$

where *T* is temperature. Reducing thermal conductivity is essential to maintain adequately large temperature difference, ΔT, between the hot and cold poles. Typical engineering TE materials exhibit *ZT* values that approach or slightly exceed 1. This relatively low value leads to device performance of about 10% of the Carnot limit, that is, about one-fourth the efficiency of conventional engines and refrigerators [12,13]. Furthermore, this implies that today’s TE devices can be employed for only limited applications in the low power regime (<500 W). To be applicable for greater power levels up to several kW, and to compete with their gas or vapor-based counterparts, increasing *ZT* values to the range of 2–3 is essential [14]. If this goal is achieved, then one promising application will be harnessing waste heat from automotive exhaust (500–800 K) to produce electricity and reduce CO_2_ emissions [15]. This poses the development of new TE materials as a grand challenge in materials science, with major implications for energy [16].

In view of the above, increasing *ZT* can be accomplished in two main directions: reducing thermal conductivity, κ, as well as increasing the electrical conductivity, σ. There are two contributions for the thermal conductivity in a lattice related to phonon vibrations, $\kappa_{p}$, and to conduction electrons, $\kappa_{e}$, so that $\kappa =\kappa_{p}+\kappa_{e}$. Herein, we will focus on increasing *ZT* by reducing the lattice thermal conductivity.

For several decades, the search for high-*ZT* materials has been conducted for single-phase materials [17], where the basic selection rules for good candidate materials are low melting temperature, large atomic masses, and large lattice parameters; however, a meager improvement from *ZT* = 0.6 to 1 has been achieved [16]. In recent years, dramatic increases in *ZT* have been achieved employing nanostructuring approaches [17,18,19,20,21,22,23,24]. The latter includes precipitation of second phases, grain refinement, mechanical alloying, and spinodal decomposition [25,26,27,28]. The underlying concept behind these methods is scattering of phonons to reduce their mean free paths, thereby reducing the lattice thermal conductivity.

The approach of embedding nanometer-size precipitates in a TE matrix has recorded successes in reducing the lattice thermal conductivity, which was computationally predicted and experimentally proven [26,29,30,31,32,33]. For example, Kim et al. investigated the role of ErAs precipitates in an In_0.53_Ga_0.47_As matrix, and predicted decrease of $\kappa_{p}$ with increasing volume fraction of ErAs [29,30]. This trend was shown experimentally for a wide temperature range up to 800 K, where the ErAs particle diameter ranges between 1 and 5 nm.

In this contribution, we will first provide a brief review of the most common approach to model the effects of a material’s microstructure on its lattice thermal conductivity, which is regularly employed for design of TE materials, Section 2. Then, we will introduce a revised and practical approach in which physical properties evaluated from first principles serve as input in a phenomenological expression for the lattice thermal conductivity, Section 3. In this revised approach we avoid the necessity of making some critical assumptions, which are demanded in the classical approach. In Section 4, we introduce implementation of the revised approach for a PbTe-matrix containing homogeneously dispersed precipitates of different size distributions, and predict the temperature-dependent lattice thermal conductivity values for different conditions. Section 5 provides a comparative analysis of the data calculated in this study.

## 2. Effects of Microstructure on Lattice Thermal Conductivity: Common Approaches

The lattice thermal conductivity is explicitly given by a simple expression derived from the kinetic theory of gases [34]:

(2) $$\kappa_{p}=\frac{1}{3}C_{v}v_{s}\lambda$$

where $C_{v}$ is the bulk constant-volume heat capacity, $v_{s}$ is the average velocity of sound in the material, and λ is the phonon mean free path. The latter is commonly expressed as $\lambda =v_{s}\tau$, where τ is the phonon relaxation time, denoting the average time between two successive phonon-scattering events. 

Phonon-scattering mechanisms usually originate from lattice defects and scattering by other phonons. The first class refers to collision of phonons with internal boundaries, impurity atoms, amorphous structures, strain fields, precipitates, or any other lattice imperfections. The second class is related to anharmonic lattice interactions, and is divided into normal (N-) processes, which are momentum-conserving, and *umklapp* (U-) processes, in which the phonon momentum is not conserved [34]. Each of the above scattering mechanisms is characterized by its own relaxation time, $\tau_{i}$, where *i* denotes the process index. Since the rate of phonon scattering in any *i*-process is proportional to the inverse of the relaxation time, ${\tau_{i}}^{-1}$, the overall effect of all processes is expressed by Matthiessen’s rule [34]:

(3) $$\tau_{t}^{-1}=\;\sum_{i}\tau_{i}^{-1}$$

The major contributions for $\tau_{t}^{-1}\;$that are usually taken into account are the N and U processes, and scattering due to internal boundaries, dislocations, strains, and precipitates; these processes are denoted by *i* = N, U, B, D, S, and P, respectively. Analytical and semi-empirical expressions for the $\tau_{i}$-values have been developed for the different *i*-processes [30]. An approximate expression for the lattice thermal conductivity, $\kappa_{p}$, as a function of $\tau_{t}\;$(depending on the relevant *i*-processes) is given by Callaway [35,36], and is commonly employed, especially for TE materials, to correlate between the material micro/nano-structure and the lattice thermal conductivity [6,26,29,30,32,33]. A useful approximation for Callaway’s expression, equivalent to expression (2), for the case where N-processes are not dominant, is the following:

(4) $$\kappa_{p}=\;\frac{k_{B}}{2\pi^{2}v_{s}}{\left(\frac{k_{B}T}{\hbar }\right)}^{3}\int_{0}^{T_{D}/T}\tau_{t}\left(x\right)\frac{x^{4}e^{x}}{{\left(e^{x}-1\right)}^{2}}dx$$

where *T_D_* is the Debye temperature, ω is the phonon angular frequency, $k_{B}$ is the Boltzmann constant, ℏ is the reduced Planck constant, and $\equiv \frac{\hbar \omega }{k_{B}T}$. For the case of *T* ≳ *T_D_*, which is usually fulfilled for semiconductors close to room temperature [34], expression (4) can be reduced to a simpler form [37]:

(5) $$\kappa_{p}\approx \;\frac{k_{B}}{2\pi^{2}v_{s}\hbar^{3}}\int_{0}^{k_{B}T_{D}}\tau_{t}\left(\omega \right)\cdot {\left(\hbar \omega \right)}^{2}d\left(\hbar \omega \right)$$

Implementation of the Callaway model for temperatures adequately higher than *T_D_* is usually performed considering the combination of U-processes and one or more of the expressions describing phonon scattering from point defects. The simple case of our interest in this contribution is a two-phase matrix/precipitates material, where the matrix is a perfect crystal with no internal boundaries or dislocations, and where no elastic strain is induced from the precipitates, so that scattering of phonons is dominated by precipitates. Additionally, for *T* ≳ *T_D_*, the dominant phonon self-scattering mechanism is the U-process [34]. We can, therefore, express $\kappa_{p}$ by employing Equation (5) once the terms $\tau_{P}$ and $\tau_{U}$ are explicitly determined [37]. For precipitate-driven scattering, $\tau_{P}^{-1}$ depends on the phonon frequency and the precipitate radius, *R*. The *near-geometrical* scattering regime for acoustic waves in solids, in analogy with electromagnetic wave scattering, applies for low frequencies and/or large precipitate radii where kR≳ 1 [37,38]; *k* is the phonon wavenumber. For this regime, $\tau_{P,G}^{-1}$ is given by [38]:

(6) $$\tau_{P,G}^{-1}=N_{v}v_{s}\left(2\pi R^{2}\right)\left[1-\frac{sin\left(2\xi \right)}{\xi }+\frac{sin^{2}\xi }{\xi^{2}}\right]$$

Here, *N_v_* is the precipitate number density (particles per unit volume) and ξ≡ kR(vsv′s−1), where $v_{s}$ and $v\prime_{s}$ are the velocities of sound in the matrix and precipitate, respectively. Note that for the particular case where the difference between e $v_{s}$ and $v\prime_{s}$ is adequately large ($\frac{v\prime_{s}-v_{s}}{v\prime_{s}}$ > 20%), expression (6) attains a simpler form:

(7) $$\tau_{P,G}^{-1}\approx N_{v}v_{s}\left(2\pi R^{2}\right)=\frac{3v_{f}}{2R}v_{s}$$

where $v_{f}$ is the precipitates’ volume fraction. The opposite extreme is the *Rayleigh* scattering regime, which applies for kR≪1. For this regime, $\tau_{P,R}^{-1}$ is given by [31,38]:

(8) $$\tau_{P,R}^{-1}=\frac{4}{9}N_{v}v_{s}\pi R^{2}{\left(\frac{\Delta \rho }{\rho }\right)}^{2}{\left(\frac{\omega R}{v_{s}}\right)}^{4}$$

where ρ is the matrix density and Δρ is the difference of densities between the matrix and precipitate. Subsequently, it was suggested that the overall relaxation time for precipitate-driven phonon scattering can be expressed in a Matthiesen-type interpolation of the scattering cross-sections associated with both mechanisms, Equations (7) and (8), which yields [31,38]:

(9) $$\tau_{P}=\tau_{P,G}+\tau_{P,R}$$

It should be remarked that the scattering cross-section for the near-geometrical regime depends on the precipitate radius only, where for the Rayleigh regime it depends also on the phonon frequency, in addition to its strong *R*^6^-dependence (typical for Rayleigh scattering).

For U-processes, the inverse relaxation time is given by [39]:

(10) τU−1≈ℏγ2Mvs2TDω2T·e−TD3T

where γ is the Grüneisen parameter, reflecting the degree of anharmonicity of lattice vibrations [34], and M is an average atomic mass of the PbTe matrix. Finally, the lattice thermal conductivity, $\kappa_{p}$, is expressed using Equation (5), employing:

(11) $$\tau_{t}={\left(\tau_{P}^{-1}+\tau_{U}^{-1}\right)}^{-1}$$

It should be noted that the result, $\kappa_{p}$, is now a function of the average radius and volume fraction of the precipitate.

## 3. Microstructure-Dependent Lattice Thermal Conductivity: A Revised Approach

The major advantage of the Callaway model is its elegant method allowing us to handle the summation of both momentum-conserving processes (namely, N-processes) with non-conserving ones (U-processes and scattering by lattice defects), whose relaxation times are not additive in a straightforward manner. To make this approach simple, the Callaway model (i) applies to elastically isotropic materials; and (ii) neglects the dispersive nature of vibrational spectrum. Also, it (iii) makes no distinction between longitudinal and transverse phonon branches, and (iv) utilizes the Planck distribution as implemented in the framework of the Boltzmann transport theory. The above assumptions pose, however, a major limitation to application of the Callaway model. Assumption (ii), based on the Debye model, means that the term $\frac{dk}{d\omega }$ of the phonon dispersion is considered to be constant for each polarization type [34]. As a result, the vibrational (phonon) density of states (v-DOS), $g_{p}\left(\omega \right)$, is a parabolic function of ω. This is, however, incorrect for most lattices. Ignoring the dispersive nature of $g_{p}\left(\omega \right)$ leads to inaccuracy in determination of $C_{v}\left(T\right)$ as well as of the effects of lattice defects on thermal conductivity, since the latter strongly depends on phonon frequency. It is noteworthy that today we have the means to calculate the full $g_{p}\left(\omega \right)$ and $C_{v}\left(T\right)$ functions accurately for a given crystal structure and symmetry, as well as evaluate $v_{s}$ for a given crystallographic orientation and a phonon branch. Assumptions (i) through (iii) become, essentially, unnecessary.

Herein, we modify the approach given in detail in Section 2 for the case of a two-phase system that includes a matrix comprising homogeneously dispersed precipitates. We first incorporate the full $g_{p}\left(\omega \right)$ and $C_{v}\left(T\right)$ functions, which are evaluated from first principles. Second, expressions (4) or (5) resulting from the Callaway model yield the lattice thermal conductivity for a specific precipitate size of an essentially δ-function size distribution. This is, however, not the case for a typical two-phase material [40], and it was shown that the precipitate size distribution has a major effect on thermal conductivity [30]. We, therefore, modify Callaway’s expression to incorporate the precipitate size distribution given by a generic φ(R)-form.

An applicable expression for the lattice thermal conductivity can be obtained directly from expression (2), noting that $\lambda =v_{s}\tau_{int}$. The magnitude $\tau_{int}$ stands for the *integral relaxation time*. Whereas $\tau_{t}\left(\omega ,R\right)$ is expressed as a function of ω and *R*, Equation (11), $\tau_{int}$ is an integral form that considers the entire spectra of ω and *R*. Also, since the number of excited phonon modes having the same ω-value is not uniform over the entire vibrational modes, then $g_{p}\left(\omega \right)$ should be taken into account in the expression for $\tau_{int}^{-1}$ as a weighting function, as follows:

(12) $$\tau_{int}^{-1}=\frac{\int_{0}^{\omega_{D}}g_{p}\left(\omega \right)\tau_{t}^{-1}\left(\omega \right)d\omega }{\int_{0}^{\infty }g_{p}\left(\omega \right)d\omega }$$

A new expression for $\tau_{t}\left(\omega \right)$ that takes into account the collective effect of the precipitates population and their size distribution will be obtained analogously to Kim et al.’s approach [29]:

(13) $$\tau_{t}^{-1}\left(\omega \right)=\;\frac{\int_{0}^{\infty }\varphi \left(R\right)\tau_{P}^{-1}\left(\omega ,R\right)dR}{\int_{0}^{\infty }\varphi \left(R\right)dR}+\;\tau_{U}^{-1}\;\left(\omega \right)$$

where $\tau_{P}^{-1}\left(\omega ,R\right)$ is given by Equation (9). For simplicity, we will further assume that φ(R) and $g_{p}\left(\omega \right)$ are normalized to unity, that is:

(14) $$\frac{1}{\omega_{D}}\int_{0}^{\omega_{D}}{g_{p}}^{\prime }\left(\omega \right)d\omega =1;\;\underset{R_{o}\to \infty }{\lim}\frac{1}{R_{o}}\int_{0}^{R_{o}}\varphi^{\prime }\left(R\right)dR=1$$

So that: $g_{p}\left(\omega \right)\equiv {g_{p}}^{\prime }\left(\omega \right)/\omega_{D}$ and $\varphi \left(R\right)\equiv \varphi^{\prime }\left(R\right)/R_{o}$. Here, $\omega_{D}$ is the Debye frequency. Substitution of expression (13) into (12) yields an expression for the integral inverse phonon relaxation time:

(15) $$\tau_{int}^{-1}=\int_{0}^{\omega_{D}}g_{p}\left(\omega \right)\left[\tau_{U}^{-1}\left(\omega \right)+\int_{0}^{\infty }\varphi \left(R\right)\tau_{P}^{-1}\left(\omega ,R\right)dR\right]d\omega$$

An expression for the thermal conductivity is, finally, obtained based on Equation (2):

(16) $$\kappa_{p}\left(T\right)=\frac{1}{3}\;C_{v}\left(T\right){v_{s}}^{2}{\left(\int_{0}^{\omega_{D}}g_{p}\left(\omega \right)\left[\tau_{U}^{-1}\left(\omega \right)+\int_{0}^{\infty }\varphi \left(R\right)\tau_{P}^{-1}\left(\omega ,R\right)dR\right]d\omega \right)}^{-1}$$

For simplicity, we keep some of the terms in expression (16) in their implicit forms. 

Most importantly, expression (16) is very practical since it enables us minimizing $\kappa_{p}$ by controlling φ(R), where the other parameters in expression (16) can be calculated. As mentioned above, the terms $g_{p}\left(\omega \right)$, $C_{v}\left(T\right)$, and $v_{s}$ can be evaluated from first principles or by other experimental means. The φ(R)-function, in turn, can be practically determined by controlling aging heat treatments [41], and quantitatively assessed applying scanning or transmission electron microscopy (SEM/TEM), as well as atom probe tomography (APT) [42,43,44]; the latter is capable of quantifying φ(R) up to a number density level of 10^21^ through 10^25^ particles/m^3^.

## 4. Implementation of the Revised Approach: The Case of Lead−Telluride (PbTe)

Herein, we will focus on PbTe-based materials. PbTe and other lead chalcogenide-based compounds, such as PbSe and PbS, are common TE materials for the mid-temperature range (600–800 K). These are narrow-gap semiconductors offering the unique combination of high Seebeck coefficient with relatively high electrical conductivity and low thermal conductivity. Owing to this combination, single-phase PbTe exhibits a maximum *ZT* value of ~0.8 [18], which can normally reach ~1.3 or surpass the limit of 2.0 under certain conditions, owing to doping and nanostructuring [22,44,45]. The lattice thermal conductivity of PbTe is ca. 2.2 W·m^−1^·K^−1^ at room temperature, and decreases with temperature in a typical 1T-dependence [46]. An example of enhancing TE properties of the PbTe-compound by introduction of a second phase is by silver additions to form Ag_2_Te precipitates dispersed in the PbTe-based solid solution [47,48,49,50,51]. Pei et al. have investigated the effects of Ag_2_Te precipitates volume fraction and average size on the thermal conductivity of PbTe for three compositions [47]. Furthermore, Lensch-Falk et al. have thoroughly investigated the morphological evolution of Ag_2_Te precipitates in PbTe matrix [51], although the effects of precipitate size distribution and their number density on thermal conductivity are yet to be researched. Additionally, vibrational properties and thermal conductivity of PbTe have been evaluated from first principles [52,53,54,55,56], as well as using molecular dynamics calculations [57]; however, incorporation of second-phase precipitates or any lattice defects other than point defects is not straightforward when applying these methods. A method combining direct calculations and analytical expressions for the effects of precipitates is, therefore, required.

### 4.1. Evaluation of Vibrational Properties from First Principles

**Figure 1:**
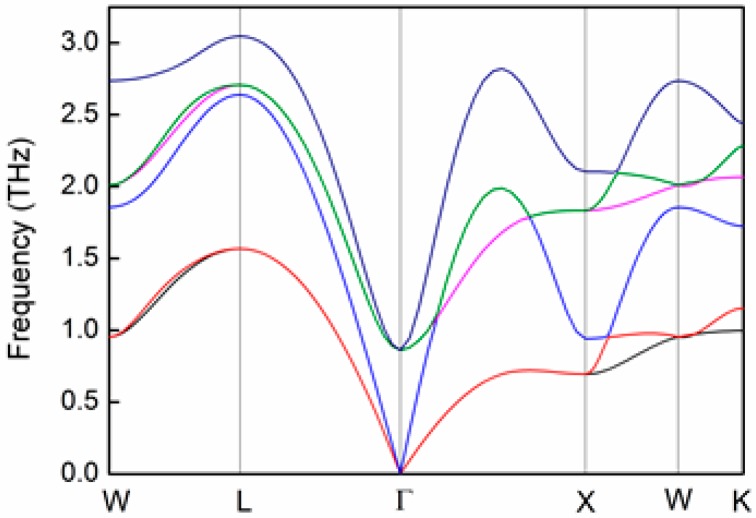
The phonon-dispersion curves of PbTe calculated from first principles for the W, L, Γ, X, and K-points of the reciprocal lattice.

We evaluate the terms $g_{p}\left(\omega \right)$, $C_{v}\left(T\right)$, and $v_{s}$ from total energy calculations employing the density functional theory (DFT) [58,59,60,61], as implemented by the Vienna *ab initio* simulation package (VASP) code [62,63,64], using the *MedeA*^®^ software environment [65]. A model PbTe lattice of the $Fm\bar{3}m$ space group symmetry is constructed and relaxed at 0 K. We utilize the general gradient approximation (GGA) to express the exchange-correlation energy, and the projector augmented wave (PAW) potentials to represent the core electrons density. The Kohn-Sham wave functions are represented using a plane-wave basis set with a 400 eV energy cutoff, and the Brillouin zone is sampled using a uniform Monkhorst–Pack *k*-point mesh with densities ranging between 0.10 and 0.15 ${Å}^{-1}$. Thresholds of 10^−6^ eV and 10^−5^ eV·${Å}^{-1}$ are set for energy convergence and Hellman–Feynman forces, respectively. The lattice parameter of PbTe obtained using these parameters is a = 6.56788 Å, in good agreement with other experimental and calculated data [54]. Vibrational calculations are performed for the relaxed structure using the direct method [66,67,68], in which inter-atomic forces are calculated by displacements of atoms within the range of ±0.02 Å with respect to their equilibrium positions, considering an interaction range of 10 Å. This provides us with the phonon-dispersion curves, v-DOS function, and temperature-dependent heat capacity. Figure 1 displays the phonon-dispersion curves for the W, L, Γ, X, and K-points.

It is indicated that both transverse modes of the acoustic phonons coincide close to the Γ-point, with sound velocity values that are significantly lower than that of the longitudinal mode. Quantitatively, the sound velocity of a given component, $v_{i}$, is determined from the pertinent acoustic mode of the dispersion curves at the Γ-point [34]:

(17) $$v_{i}={\frac{d\omega }{dk_{i}}|}_{k\to 0}$$

The average sound velocity, $v_{s}$, is evaluated as a harmonic average of the one longitudinal and the two transverse components of sound velocity, $v_{L}$, $v_{T1}$, and $v_{T2}$, respectively [67,69]:

(18) $$v_{s}={\left[\frac{1}{3}\left(v_{L}^{-3}+v_{T1}^{-3}+v_{T2}^{-3}\right)\right]}^{-\frac{1}{3}}$$

**Figure 2:**
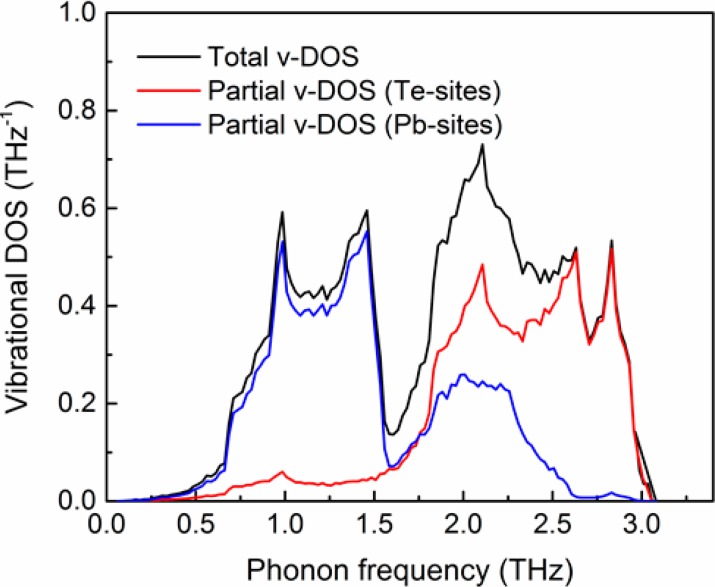
The vibrational density of states (v-DOS) of PbTe calculated from first principles. The total v-DOS appears in black, and the partial v-DOS of the Te- and Pb- sublattice sites appear in red and blue, respectively.

The sound velocity components derived from the data in Figure 1 are $v_{L}$ = 3570.5 m·s^−1^ and $v_{T1}$ = $v_{T2}$ = 1210.5 m·s^−1^. Accordingly, the average sound velocity is evaluated to be $v_{s}$ = 1376.8 m·s^−1^. The v-DOS, $g_{p}\left(\omega \right)$, is calculated based on the data shown in Figure 1, and is plotted in Figure 2 (black curve), together with the partial v-DOS associated to the Te-sites (red) and Pb-sites (blue).

**Figure 3:**
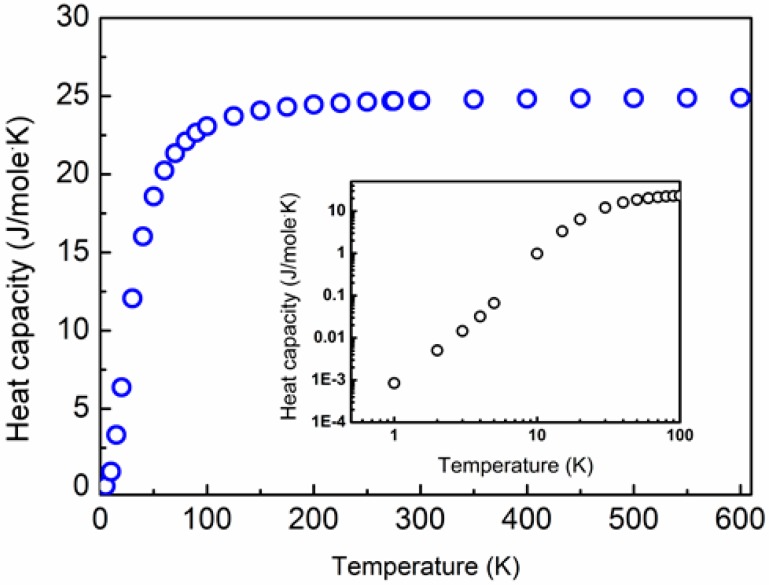
The temperature-dependent heat capacity, $C_{v}\left(T\right)$, of PbTe as calculated from first principles, applying the Debye approximation, for the temperature range of 0 through 600 K. Inset: a double-logarithmic plot showing the linear dependence of $C_{v}\left(T\right)$ on T^3^ for temperatures below 20 K.

Finally, the temperature-dependent heat capacity, $C_{v}\left(T\right)$, is calculated based on the $g_{p}\left(\omega \right)$ function applying the Debye approximation, and is plotted in Figure 3 for the temperature range of 0 through 600 K.

Additional parameters required to evaluate $\kappa_{p}\left(T\right)$ based on Equation (16) are the Debye temperature and Grüneisen parameter of the PbTe-matrix. They are evaluated as *T_D_* = 136 K and *γ* = 1.96, respectively, based on the thorough study by Zhang et al. [56], which were implemented for PbTe [26,32,70].

### 4.2. Effects of Precipitates on Lattice Thermal Conductivity

In this section we apply our revised approach to evaluate the temperature-dependent lattice thermal conductivity, and how it is affected by the precipitate average radius (*R*), precipitate size distribution (characterized by ∆*R*), and precipitate number density (N_v_) or volume fraction ($v_{f}$). Herein, we apply expression (16) as well as input values calculated from first principles, namely $g_{p}\left(\omega \right)$, $C_{v}\left(T\right)$, and $v_{s}$, which are given in Section 4.1. To represent the finite size distribution of the precipitates, we utilize a Gaussian distribution, given explicitly by:

(19) $$\varphi \left(R\right)=\frac{1}{\sqrt{2\pi }\cdot \Delta R}e^{\;-\frac{{\left(R-R_{o}\right)}^{2}}{2{\left(\Delta R\right)}^{2}}}$$

Expression (19) fulfills the normalization condition given by (14), where *R_o_* and ∆*R* represent the average radius and standard deviation of the R-distribution, respectively. We use the term ∆*R* to denote the distribution ‘width’. We note that a log-normal distribution is usually typical for precipitates formed naturally in aging processes; however, the former one is more simple to handle, and is adequately accurate for R-values close to the average radius.

**Figure 4:**
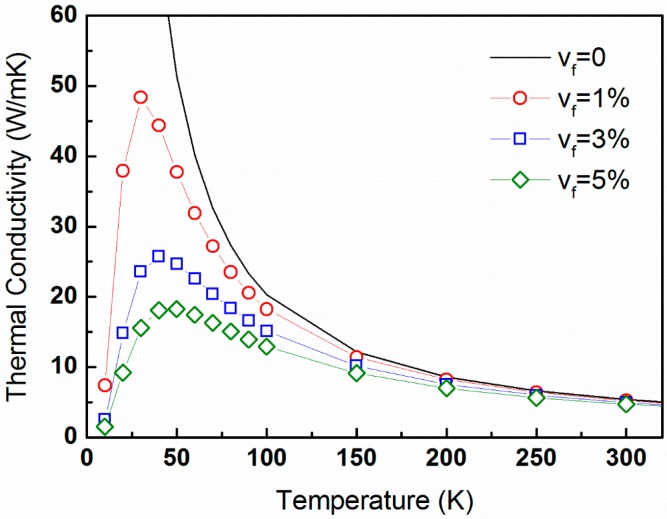
The temperature-dependent lattice thermal conductivity of PbTe calculated from expression (16) for the temperature range of 0 through 300 K and a constant precipitate size distribution characterized by *R_o_* = 30 nm and ∆*R* = 5 nm, and for volume fractions of 0%, 1%, 3%, and 5% denoted by the black line, red circles, blue squares, and green diamonds, respectively.

Figure 4 displays the temperature-dependent lattice thermal conductivity calculated from expression (16) for the temperature range of 0 through 300 K and a constant precipitate size distribution characterized by *R_o_* = 30 nm and ∆*R* = 5 nm, and for volume fractions of 0%, 1%, 3%, and 5%. The latter are equivalent to precipitate number density values of 0, 8.84 × 10^19^, 2.65 × 10^20^, and 4.42 × 10^20^ m^−3^, respectively.

**Figure 5:**
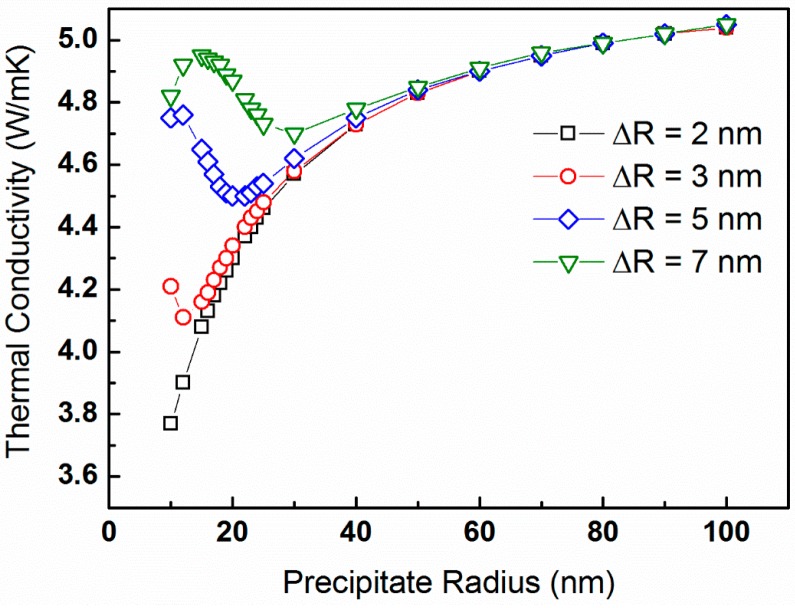
The lattice thermal conductivity of PbTe calculated from expression (16) for constant temperature of *T* = 300 K and precipitate volume fraction of $v_{f}$= 5% as a function of the average precipitate radius. The calculations are for precipitate size distributions of ∆*R* = 2, 3, 5, and 7 nm, denoted by black squares, red circles, blue diamonds, and green down-triangles, respectively.

As expected, the lattice thermal conductivity generally decreases with increasing precipitate volume fraction. We calculate the lattice thermal conductivity for a constant temperature of *T* = 300 K and precipitate volume fraction of $v_{f}$ = 5% as a function of the average precipitate radius, which ranges between 10 and 100 nm. The latter correspond to precipitate number density values of N_v_ = 1.19 × 10^22^ through 1.19 × 10^19^ m^−3^, respectively. These $\kappa_{p}$-values are plotted in Figure 5 for precipitate size distributions of ∆*R* = 2, 3, 5, and 7 nm.

A general trend of $\kappa_{p}$ increasing with increasing *R_o_* is apparent, with deviations for large ∆*R*-values, pointing on the essence of precipitate size distribution. 

**Figure 6:**
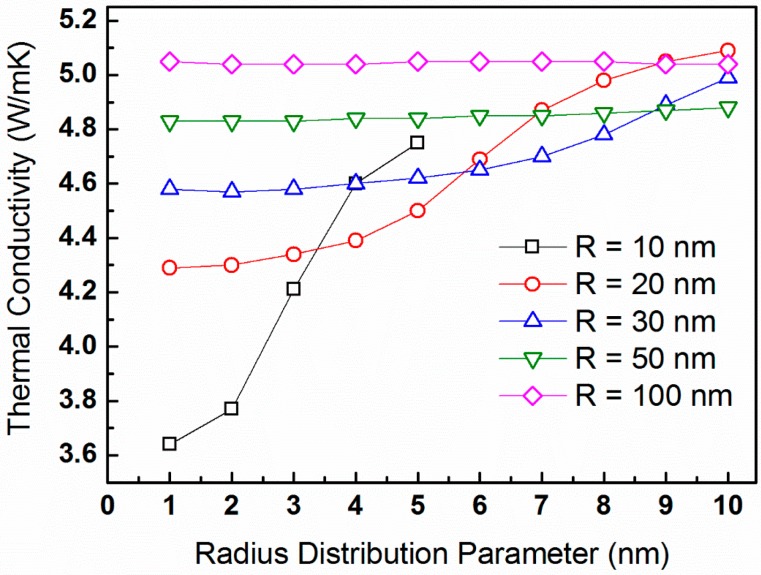
The lattice thermal conductivity of PbTe calculated from expression (16) for constant temperature of *T* = 300 K and precipitate volume fraction of $v_{f}$ = 5% as a function of the ∆*R*-parameter. The calculations are for average radii of *R_o_* = 10, 20, 30, 50, and 100 nm, denoted by black squares, red circles, blue upward triangles, green downward triangles, and magenta diamonds, respectively.

To further demonstrate the effects of size distribution, we calculate the lattice thermal conductivity for a constant temperature of *T* = 300 K and precipitate volume fraction of $v_{f}$ = 5% as a function of the ∆*R*-parameter ranging from 1 through 10 nm. Figure 6 plots $\kappa_{p}$ for the average radii of *R_o_* = 10, 20, 30, 50, and 100 nm, which correspond to N_v_-values of 1.19 × 10^22^, 1.49 × 10^21^, 4.42 × 10^20^, 9.95 × 10^19^, and 1.19 × 10^19^ m^−3^, respectively.

The lattice thermal conductivity is, apparently, independent of the precipitate size distribution for average radii of 50 nm or larger. For smaller precipitate radii, lattice thermal conductivity increases with the increase of ∆*R*-parameter.

**Figure 7:**
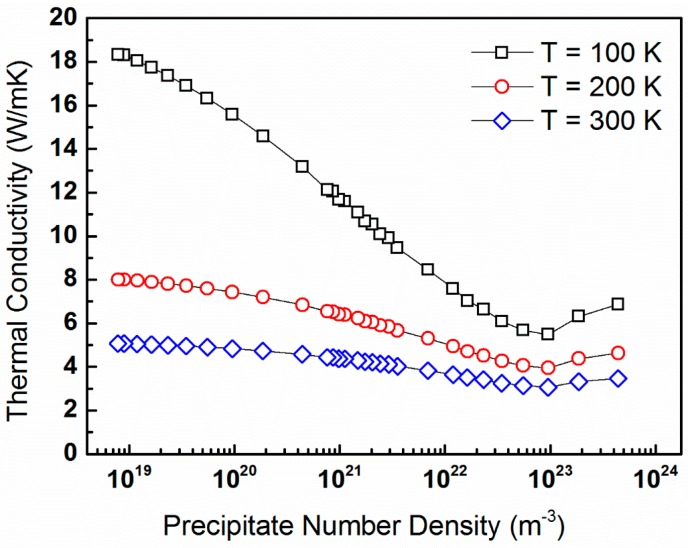
The lattice thermal conductivity of PbTe calculated from expression (16) as a function of the precipitate number density for $v_{f}$ held constant at 5% and ∆*R* = 1 nm, for *T* = 100 (black squares), 200 (red circles), and 300 K (blue diamonds).

A different way to consider the effects of precipitates on lattice thermal conductivity is to examine its dependence on number density. Figure 7 displays the lattice thermal conductivity of PbTe as a function of the precipitate number density for $v_{f}$ held constant at 5% and ∆*R* = 1 nm, for *T* = 100, 200, and 300 K.

An expected trend is observed, in which $\kappa_{p}$-values generally decrease with increasing N_v_, up to a value of ca. 10^23^ m^−3^, which corresponds to *R_o_* ≈ 5 nm.

**Figure 8:**
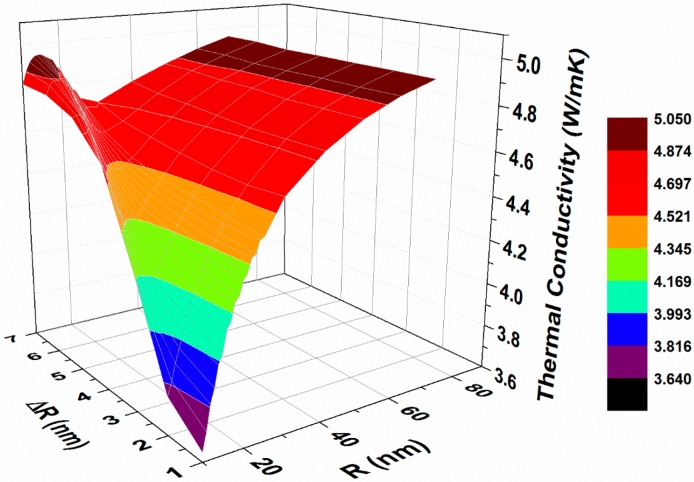
The lattice thermal conductivity of PbTe calculated from expression (16) as a function of the precipitate average radius and size distribution for *T* = 300 K and $v_{f}$ = 5%.

Figure 8 summarizes the combined effects of precipitate average radius and size distribution on lattice thermal conductivity for *T* = 300 K and $v_{f}$ = 5%.

As expected, for large values of *R_o_*, the influence of ∆*R* is negligible. For radii smaller than 30 nm, the trend is more complicated.

### 4.3. Effects of Matrix Composition on Lattice Thermal Conductivity 

**Figure 9:**
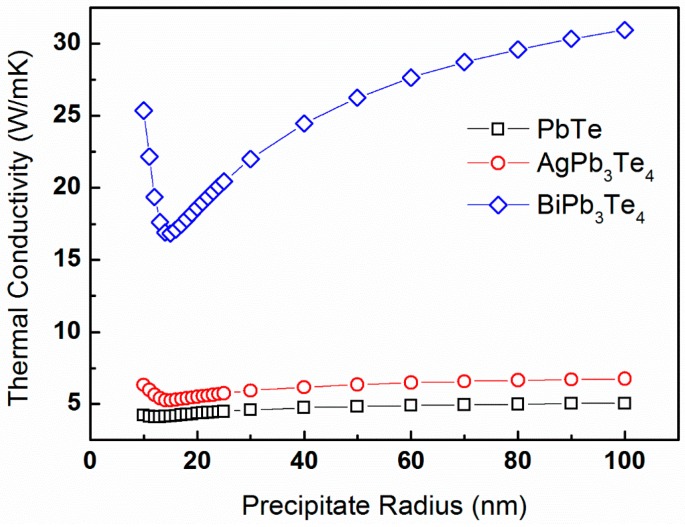
The lattice thermal conductivity of PbTe calculated from expression (16) for constant temperature of *T* = 300 K, precipitate volume fraction of $v_{f}$ = 5%, and Δ*R* = 2 nm as a function of the average precipitate radius. The calculations are for the PbTe, AgPb_3_Te_4_, and BiPb_3_Te_4_ compounds, denoted by black squares, red circles, and blue triangles, respectively.

In this section, we study the effects of matrix composition, as manifested by $g_{p}\left(\omega \right)$ and $v_{s}$, on the lattice thermal conductivity for different R_o_- and ∆R-values. To this end, we construct two model compounds in which Ag- or Bi-atoms substitute for the Pb-sublattice sites of PbTe. The model compounds simulated are AgPb_3_Te_4_ and BiPb_3_Te_4_, for which we perform the same DFT calculations described in Section 4.1. To differentiate the effects of $g_{p}\left(\omega \right)$ and $v_{s}$ from the other factors, we assume that the values *T_D_* = 136 K and *γ* = 1.96 are the same as for the PbTe model compound. The average sound velocities evaluated for these compounds are $v_{s}$ = 1420 and 2039 m·s^−1^, respectively. Applying the aforementioned routine, we calculate the lattice thermal conductivity for a constant temperature of T = 300 K, precipitate volume fraction of $v_{f}$ = 5%, and ΔR = 2 nm as a function of the average precipitate radius, which ranges between 10 and 100 nm. The latter corresponds to precipitate number density values of N_v_ = 1.19 × 10^22^ through 1.19 × 10^19^ m^−3^, respectively. These $\kappa_{p}$-values are plotted in Figure 9 for the PbTe, AgPb_3_Te_4_, and BiPb_3_Te_4_ compounds.

**Figure 10:**
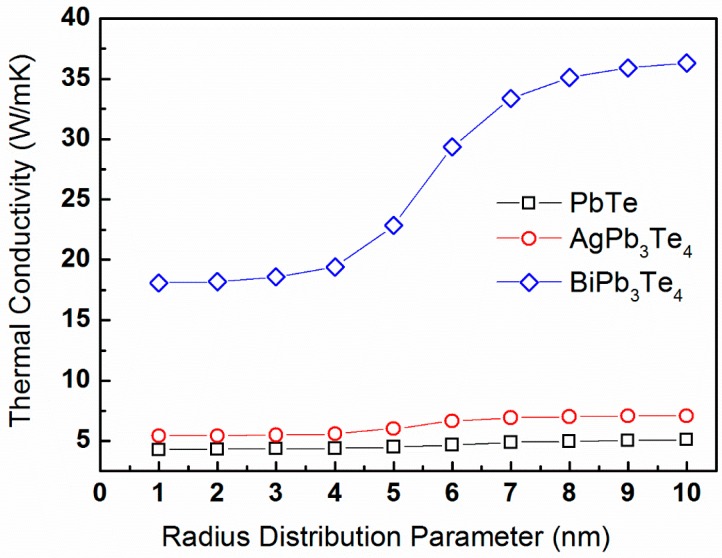
The lattice thermal conductivity of PbTe calculated from expression (16) for constant temperature *T* = 300 K, average radius *R_o_* = 20 nm, and precipitate volume fraction of $v_{f}$ = 5% as a function of the ∆*R*-parameter. The calculations are for the PbTe, AgPb_3_Te_4_, and BiPb_3_Te_4_ compounds, denoted by black squares, red circles, and blue triangles, respectively.

Trends similar to those shown in Figure 5 are apparent. A significant difference between the $\kappa_{p}$-values obtained for the three compounds is observed. The effects of precipitate size distribution on lattice thermal conductivity are different for the three compounds simulated. Figure 10 displays plots of $\kappa_{p}$-values calculated for a constant temperature of *T* = 300 K, *R_o_* = 20 nm, and precipitate volume fraction of $v_{f}$= 5% as a function of the ∆*R*-parameter, ranging from 1 through 10 nm.

It is indicated that the lattice thermal conductivity increases with ∆*R*-values, and this trend is mostly prominent for the BiPb_3_Te_4_-model compound.

## 5. Discussion

There are many studies dealing with effects of second-phase precipitation on thermal conductivity of TE compounds, in particular for PbTe-based compounds [22,26,32,44,45,46,47,70,71,72,73]. First, our room temperature value of calculated lattice thermal conductivity, ca. 5 W·m^−1^·K^−1^, is in good agreement with such values reported in literature for similar conditions [22,32]. Other values reported in literature for PbTe-based compound at room temperature are lower, ca. 3.0 through 4.5 W·m^−1^·K^−1^ [26,44,45,47,70,72]. This difference can be associated with the variety of impurity levels prevailing at the PbTe-matrix for the experimentally investigated materials, whereas our calculations are valid for an ideally pure PbTe-matrix. Second, the trends predicted by our calculations indicate reduction of lattice thermal conductivity with increasing precipitate number density and volume fraction by a few tens of percent (depending on temperature), which is also implied by other reports [22,26,32,44,45,47,70,72]. We note, however, that the latter effects are more difficult to compare, since they are complicated and strongly depend on experimental conditions. For example, effects of precipitates and solute atoms at the matrix are hardly resolvable from each other, as is well demonstrated by Zhao et al. [21], Tan et al. [22], and Heinz et al. [74].

Notwithstanding the above thorough studies on precipitation in PbTe, effects of precipitate size distribution are seldom reported. This can be associated with experimental challenges in synthesis of a system comprising controllable precipitate size distribution [75]. A remarkable effort toward realization of the effects of particle size distribution on phonon scattering was reported by Kim and Majumdar, in which particles of Γ-size distribution were treated in an analytical model, and an expression for the scattering cross-section considering both Rayleigh and near-geometrical regimes was developed [38]. This approach, however, was not incorporated in an analytic expression for lattice thermal conductivity and could not be tailored to a matrix having a given v-DOS pattern. The essence of the present study lies in establishment of a coupling factor between the $g_{p}\left(\omega \right)$- and φ(R)-functions, as featured by expression (12). This originates from the intensity of interaction of phonons with precipitates, which depends on the precipitate size, where large precipitates scatter low-frequency phonons more efficiently and vice versa. 

The data shown in Section 4.2 and Section 4.3 indicate that lattice thermal conductivity generally decreases with increasing precipitate volume fraction, Figure 4. Also, the general trend of $\kappa_{p}\left(T\right)$ decreasing with temperature is apparent for elevated temperatures due to U-processes, which are expressed in Equation (10). This trend, shown in Figure 4, is balanced by low values of heat capacity as well as by processes of phonon scattering by precipitates, as featured in Equation (8), which thus predominate at low temperatures (<50 K). This balancing mechanism is manifested by relaxation times for U-processes that increase with decreasing temperature, and surpass the relaxation times for phonon scattering by precipitates. This crossover occurs at characteristic relaxation times of ca. 10^−10^–10^−9^ s. Simultaneously, the decrease of heat capacity with decreasing temperatures is typical for low temperatures. This is clearly observed in the inset of Figure 3, where $C_{v}\left(T\right)$ is plotted on a double-logarithmic scale, signifying the well-known linear dependence of $C_{v}\left(T\right)$ on T^3^ [34,67,68].

For the case in which the precipitate volume fraction is held constant, Figure 5, we observe a general trend of $\kappa_{p}$ increasing with increasing *R_o_*, which is reasonable. This is because the number density of precipitates also increases, given that the latter serve as phonon-scattering centers. For low values of *R_o_*, deviations from this trend are observed, and they become more significant for large ∆*R*-values. This signifies the importance of precipitate size distribution. The sensitivity of lattice thermal conductivity to the precipitate size distribution is prominent in Figure 6, particularly for average radii as small as 30 nm. Figure 7 reveals an expected trend, in which $\kappa_{p}$-values generally decrease with increasing N_v_, up to a value of ca. 10^23^ m^−3^, which corresponds to *R_o_* ≈ 5 nm. Such observation was reported by us earlier [68]. For precipitate radii smaller than that, the term ∆*R* becomes critical. It is also shown that the dependence of $\kappa_{p}$ on N_v_ is stronger for lower temperatures [76]. Interestingly, trends similar to those shown in Figure 7 are reported by Mingo et al. [31], who applied an analytic approach based on the Callaway model to simulate the effects of nanoparticles on lattice thermal conductivity of SiGe alloys. They calculated the lattice thermal conductivity as a function of particle radius for particles of different materials and with a constant 0.8 vol %, and found that minimum thermal conductivity values are attained for an optimum radius ranging between 2 and 5 nm, depending on the material. For these conditions, increase of particle radius is equivalent to decrease of number density, so that the optimum values range between N_v_ = 2.4 × 10^23^ and 1.5 × 10^22^ m^−3^, respectively. This reasonably agrees with the data shown in Figure 7. Similar trends are introduced in a theoretical study of SiGe alloys reported by Kundu et al. [77].

Figure 8 shows that the influence of ∆*R* is negligible for large values of *R_o_*, whereas for radii smaller than 30 nm, the trend is more complicated. For this regime, the data shown in Figure 8 can serve as guidelines for design of the matrix/precipitate system with optimized $\kappa_{p}$, by selecting the appropriate heat treatments [41]. Such optimization rests upon the concept that the desirable φ(R)-function is tailored for a given TE matrix with a given v-DOS. We note that a relatively simple form of the φ(R)-function was chosen in order to use the parameter ∆*R* to represent the significance of the precipitate size distribution. We believe that an alternative functional form of φ(R) should have yielded similar qualitative conclusion.

The effects of matrix composition are apparent in Figures 9,10, considering the different results obtained for the three PbTe, AgPb_3_Te_4_, and BiPb_3_Te_4_ model compounds. First, Figure 9 indicates trends similar to those shown in Figure 5. The distinct difference between the $\kappa_{p}$-values obtained for the three compounds is probably associated with the difference in sound velocity as well as v-DOS. Second, similar to the results shown in Figure 6, the lattice thermal conductivity is sensitive to the precipitate size distribution, particularly for large ∆*R*-values. The BiPb_3_Te_4_-compound exhibits such sensitivity more prominently compared to its two counterparts, which are associated with higher sound velocity values and different v-DOS. To distinguish between the roles of sound velocity and v-DOS, we calculate the ratio of squared sound velocities and compare it to the ratio of lattice thermal conductivities for identical *R_o_*-values and at 300 K, which is adequately larger than *T_D_*. Based on Equation (16), identical v-DOS values should yield identical ratios. Any difference between both ratios will be associated with difference in the v-DOS functions. We evaluate a ratio of $v_{s}^{2}$(BiPb_3_Te_4_) $v_{s}^{2}$(AgPb_3_Te_4_):$v_{s}^{2}$(PbTe) ≈ 2.20:1.06:1; and $\kappa_{p}$(BiPb_3_Te_4_):$\kappa_{p}$(AgPb_3_Te_4_):$\kappa_{p}$(PbTe) ≈ 36.32:7.1:5.09 (based on Figure 10). This means that the major contribution to the increased $\kappa_{p}$-values observed for the BiPb_3_Te_4_ model compound with respect to that of PbTe is due to different v-DOS functions, whereas the difference between the $\kappa_{p}$-values of AgPb_3_Te_4_ and PbTe is more likely due to the difference in sound velocities. 

The effects of Ag- and Bi-substitutions for the Pb-sublattice sites of PbTe on its lattice thermal conductivity can be understood in view of the v-DOS spectrum shown in Figure 2. The low-frequency part of the v-DOS spectrum (<ca. 1.7 THz) is associated with Pb-sublattice vibrations, whereas the higher-frequency part belongs to Te vibrations. This was also reported by Pereira et al., based on neutron-inelastic scattering [78], as well as by Qui et al., based on molecular dynamics calculations [57]. Since this regime is dominated by acoustic phonons, we expect that variations of the v-DOS for frequencies lower than ca. 1.7 THz should affect $\kappa_{p}$ more significantly. This well corresponds with the explanations provided by He at al. [32]. In this context, we note that the v-DOS calculated by us, Figure 2, exhibits similar behavior to those reported by these authors in terms of the distinction between two main branches, namely the low- and high-frequency regimes, as well as the entire frequency range of v-DOS, that is, up to 3.6–4.2 THz; for comparison, our v-DOS spectrum extends up to ca. 3 THz. The phonon-dispersion curves and temperature-dependent heat capacity calculated by us (Figure 1,Figure 3 , respectively) correspond with those reported by Zhang et al. [56], Tian et al. [79], and Romero et al. [52,53]. We note that the value of $v_{s}$ = 1376.8 m·s^−1^ calculated by us for PbTe based on Equations (17) and (18) are relatively low with respect to other computational [54,55] and experimental [26,32,70,78] results reported in thee literature. This can be correlated to the method applied in this study, in which sound velocity components are derived from phonon-dispersion curves, as explained by us elsewhere [68].

## 6. Summary and Conclusions

We establish a practical approach to evaluate the temperature dependence of lattice thermal conductivity, $\kappa_{p}\left(T\right)$, in a system of matrix containing homogeneously dispersed precipitates. This approach applies vibrational properties that are evaluated from first principles, namely the average sound velocity, vibrational density of states, and heat capacity, as well as a modified expression for the phonon relaxation time. The latter term rests upon the concept that phonon-scattering efficiency depends on both phonon frequency and precipitate radius, in a way that high-frequency phonons are scattered more effectively from small precipitates, and vice versa. This yields an expression that utilizes the vibrational density of states as a weighting function for a certain precipitate population having any size distribution function.

In this study, we implement our approach for a lead−telluride (PbTe) thermoelectric (TE) matrix comprising precipitates with Gaussian size distribution, characterized by an average radius of *R_o_* and a standard deviation ∆*R*. For simplicity, these precipitates have no chemical identity. We find that $\kappa_{p}\left(T\right)$ generally decreases with the increase of precipitate volume fraction. The expected trend, in which $\kappa_{p}$ decreases with temperature, is apparent for elevated temperatures due to U-processes. It is balanced at low temperatures by low values of heat capacity as well as by processes of phonon scattering by precipitates, which predominate at low temperatures (<50 K). We also observe a general trend of $\kappa_{p}$ increasing with increasing *R_o_* for constant precipitate volume fractions. For low values of *R_o_*, deviations from this trend are observed, and they become more significant for increasing ∆*R*-values; this highlights the essence of precipitate size distribution. The sensitivity of lattice thermal conductivity to the precipitate size distribution is prominent for average radii as small as 30 nm. Quantitatively, $\kappa_{p}$-values generally decrease with increasing precipitate number density, N_v_, up to a value of ca. 10^23^ m^−3^, which corresponds to *R_o_* ≈ 5 nm. It is also shown that the dependence of $\kappa_{p}$ on N_v_ is stronger for lower temperatures, and the effects of lattice defects diminish with increasing temperatures. 

The data reported in this study can serve as guidelines for the design of TE matrix/precipitate systems with optimized $\kappa_{p}$, e.g., by selecting the appropriate heat treatments, in a way that the precipitate size distribution can be tailored for a matrix with given vibrational properties.
